# Reducing first appointment delays for electron radiotherapy patients by improving the treatment planning pathway: a quality improvement project

**DOI:** 10.1136/bmjoq-2022-002221

**Published:** 2023-11-21

**Authors:** Louise Gately, Katie Sanders, Nathan Proudlove

**Affiliations:** 1 Medical Physcis Department, Clatterbridge Cancer Centre NHS Foundation Trust, Liverpool, UK; 2 Alliance Manchester Business School, The University of Manchester, Manchester, UK

**Keywords:** radiotherapy, PDSA, process mapping, statistical process control, quality improvement

## Abstract

Clatterbridge Cancer Centre (CCC) is a specialist hospital trust in England with three sites.

Delay to the start of an appointment for radiotherapy, especially the first appointment (a ‘New Start’) is poor, both for operational efficiency and patient experience, causing stress for both patients and staff. Our aim is for the New Start to begin within 30 min of the allotted appointment time. To this end, we established another aim: for ‘Final Checks’ to the radiotherapy plan to be completed at least 30 min prior to the New Start appointment time.

Prior to this quality improvement (QI) project, only 33% of electron-treatment New Start appointments started within the target 30 min (the average delay was 52.4 min) and only 48% of the corresponding Final Checks had been completed by their 30 min prior target.

The treatment pathway for these patients was redesigned, with the aim of 90% of New Start appointments starting within 30 min of the allotted appointment time.

By the end of this QI project, 69.2% of New Start appointments started within 30 min of the appointment time (with average delay reduced to 27.2 min), and 92.3% of Final Checks were completed by their 30 min prior target. We also reduced the number of safety (Datix) incidents due to plan not ready from 10 to 0. A year after the project, we have held most of the time improvements and still have had 0 plan-not-ready Datix.

The largest improvement was achieved by introducing a proxy (without the patient present) ‘day 0’ appointment. This takes place in advance of the New Start appointment to enable earlier planning. Subsequent improvements included: automating previously manual planning calculations, making the care path consistent with other external beam radiotherapy care paths at CCC to reduce staff cognitive load and sharing key performance data with staff.

**WHAT IS ALREADY KNOWN ON THIS TOPIC**

- The literature confirms our experience that patients treated with electron radiotherapy experience longer delays than other external beam radiotherapy (EBRT) patients. The literature contains examples of application of quality improvement (QI) thinking to other types of EBRT.

**WHAT THIS STUDY ADDS**

- To our knowledge, this is the first application of QI thinking to electron radiotherapy planning in the literature. Despite the relatively small number of electron patients treated at Clatterbridge Cancer Centre, this QI project demonstrates that it is possible to make meaningful and sustained reductions in delays experienced by patients at their first fraction, also eliminating consequent Datix (safety) incidents.

**HOW THIS STUDY MIGHT AFFECT RESEARCH, PRACTICE OR POLICY**

- This study demonstrates that QI thinking can be applied successfully to a low-throughput radiotherapy treatment planning pathway.

## Problem

The Clatterbridge Cancer Centre (CCC) National Health Service (NHS) Foundation Trust is one of the UK’s regional tertiary (specialist) hospital trusts. We have three sites in the northwest of England: in Liverpool city centre and its suburb of Aintree, and across the River Mersey at Clatterbridge on the Wirral. CCC provides highly specialized cancer care to both inpatients and outpatients, including pioneering chemotherapy, immunotherapy, gene therapy, haemato-oncology and radiotherapy. Our catchment is 2.4 million people across Cheshire, Merseyside and the surrounding areas, including the Isle of Man.^1^

Radiotherapy uses ionising radiation to kill or shrink cancerous tumours. The most common technique is to direct a beam of photons (mega voltage (MV) X-rays or gamma rays) or subatomic particles (electrons or protons) from outside the body using a linear accelerator (linac) or (for protons) a cyclotron. (The alternative to external beam radiotherapy (EBRT) is to place a radiation source inside the body, eg, brachytherapy.^2^) Most EBRT is photon therapy, but a small number of patients receive electron beam therapy (or, for very few, proton beam therapy).^3^ We have 10 linacs across our three sites, 2 of which are commissioned to provide electron radiotherapy. In 2021, we administered 728 electron treatments (fractions).

At CCC, all patients receiving MV photon therapy have a CT scan to aid planning. However, the majority of our electron patients do not, since clinicians deem it unnecessary in most cases; this group is described locally as non-scanned electron patients—this is the target group for our quality improvement (QI) project, referred to simply as ‘patients’ from now on. These make up approximately 1% of our total treatment workload at CCC.

Prior to this improvement project, our electron treatment planning care pathway had a number of tasks which required manual calculations and manual data entry. The small patient numbers meant that these tasks were unfamiliar to staff, the tasks having been automated on other EBRT pathways to improve patient safety. As a result, these manual tasks caused delays, producing significant time pressures at the patient’s New Start—their appointment for their first radiotherapy treatment (first radiation fraction). Further stress and delays at the New Start arose from having to determine the required setup parameters while the patient was present and adjust the treatment plan accordingly. Amending the treatment plan, at this stage and under pressure, can also introduce inadvertent errors.

The primary aim of this project was to reduce delays at New Start, and so improve the experience for both patients and staff. We hoped that as a result of changes, 90% of New Start appointments would commence within 30 min of their allotted time by August 2021, a substantial improvement over the previous level of 33%. There would be consequent secondary impacts: reducing staff stress and increasing patient safety.

We used the Model for Improvement (MfI) and its Plan–Do–Study–Act (PDSA) cycles.^4–6^ This approach has been used successfully in other NHS clinical science areas: life sciences^7–10^ and physiological sciences.^11^

## Background

The majority of radiotherapy patients receive photon EBRT for the treatment of deep-seated tumours; however, for a subset of patients with superficial (shallow) tumours, electron EBRT is a more suitable treatment.^12^ The majority of these patients have non-melanoma skin cancer, which often appears on sun-exposed areas of skin such as the head, neck and other extremities, although any superficial tumour can be treated with electrons.^12^

For such superficial tumours, the advantage of electrons over photons is that they provide a high dose at the skin, followed by rapid dose fall-off deeper into the body (beyond the tumour).^12^ Tissue-equivalent material may be placed on top of the tumour to increase the skin dose to the desired level—usually 90–95% of the prescribed dose.^12^ Electrons are often used to treat lesions between 1 and 5 cm deep with a single radiation field; treatment is relatively simple compared with photons.^12^ The lesions are, by their nature, superficial and can be palpated and marked up in clinic, so our clinicians often choose not to authorise a CT planning scan for these patients.

**Figure 1:**
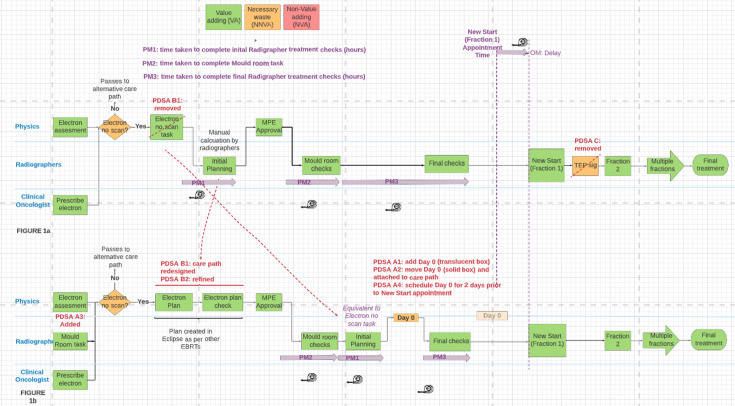
Process maps: (A) initial and (B) final. EBRT, external beam radiotherapy; MPE, medical physics expert; OM, outcome metric; PDSA, Plan–Do–Study–Act; PM, process metric; TEP Sig, treatment expert practitioner sign-off.

The majority (95%) of our electron patients receive treatment to either their head and neck or to their extremities, and it is policy at CCC to immobilise these patients in a thermoplastic cast during treatment to limit movement. (Other common body radiation treatment sites are the breast and sternum for which the patient does not have a cast.) The cast is made and checked in the Mould Room as part of the planning pathway, as shown in figure 1.

Previously, there had been numerous incremental changes to this patient care pathway. For example, in 2017, a paperless process was introduced, which improved both efficiency and safety (through staff familiarity, since all our other EBRT treatments were already paperless).

In 2019, further changes were made, including the introduction of plan approval by a medical physics expert (MPE)^13^ again to improve safety and bring this pathway into line with other EBRT treatments, all of which have either plan approval by the responsible clinician or an MPE under a clearly defined protocol.^14^ While this meant patient plans were approved within the ARIA radiation therapy management record-and-verify software system,^15^ it also meant that these approved patent plans had to be ‘unapproved’ to allow the final treatment parameters to be captured and added.

This pathway is different from all other types of EBRT, where patients have a CT scan. The CT scan allows appropriate treatment parameters (gantry, collimator and couch positions) to be determined at the planning stage. Non-scanned electron patients do not have a CT scan, so a treatment simulation has to be performed to determine setup positions while the patient is on the radiotherapy couch at their New Start appointment. To enable the newly determined treatment parameters to be captured, the previously approved plan needs to be unapproved. This unapproval with the patient on the bed causes stress and concern to staff. Time pressures caused by delays earlier in the planning pathway add to this stress. The consequences were delays at New Start plus 13 events (10 of which were plan not ready) over 21 patients requiring recording in our patient safety incident reporting system (Datix).^16 17^ These delays are consistent with the literature which demonstrates that electron patients experience longer delays than other EBRT patients.^18^

Similar QI projects specifically related to radiotherapy planning are rare. In cervical cancer treatment, a QI project reduced planning times for brachytherapy^19^ and another increased the proportion of cases treated within a target total time for prolonged chemoradiation.^20^ Price *et al* suggest how routinely collected data on healthcare outcomes could be used in a PDSA-type way to iterate towards better trade-offs between lung tumour reduction and sparing collateral damage to the heart.^21^

## Measurement

For this project, we established a QI team comprising medical physicists and both pretreatment and treatment radiographers. Initially, a process map was developed from care paths recorded within ARIA. Figure 1A shows all steps in this part of the pathway. As shown, there are three teams responsible for tasks: physicists, radiographers and clinical oncologists. During previous lean-style waste removal^6^ work on this pathway, many non-value adding steps^22^ had been addressed. However, there were some still necessary non-value adding steps,^23^ shaded orange, that needed to be further investigated. These were: the decision point for planning/no planning (‘Electron No Scan?’) and the treatment expert practitioner (TEP) sign-off (‘TEP Sig’).

**Figure 2:**
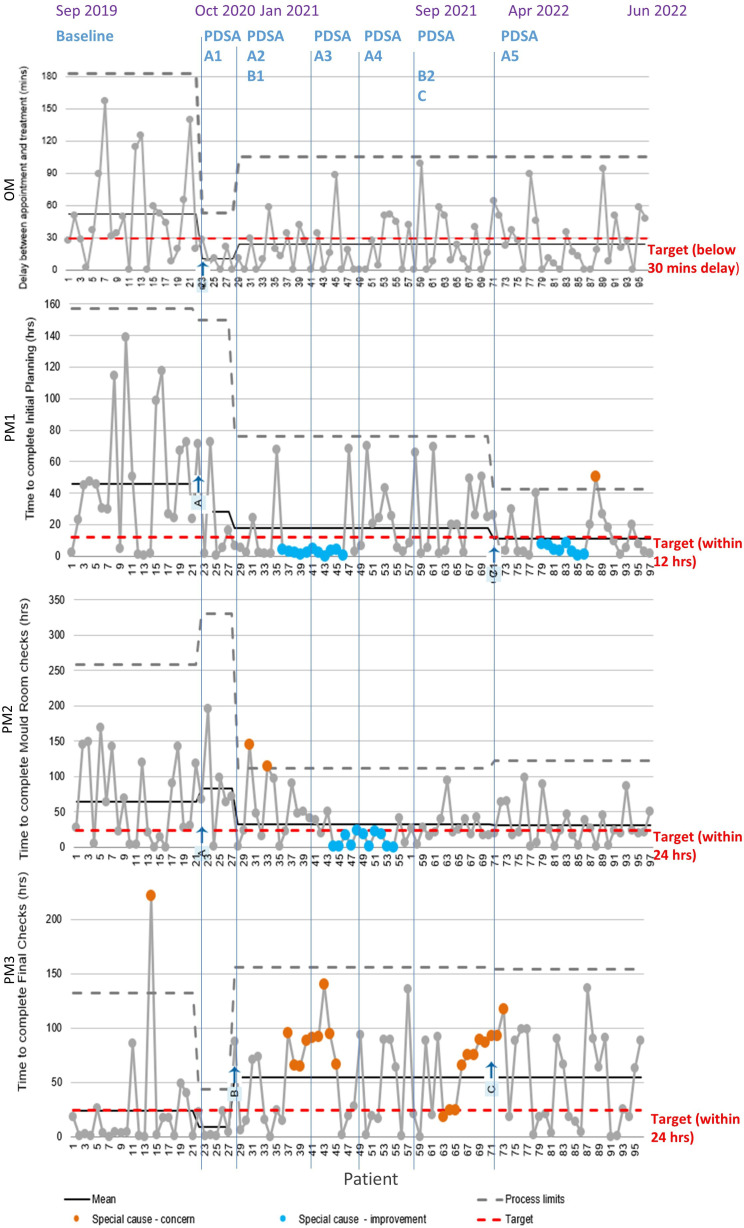
Performance metrics over baseline and PDSA cycles. OM, outcome metric; PDSA, Plan–Do–Study–Act; PM, process metric.

To assess whether changes constituted an improvement, we established a set of metrics and analysed them using statistical process control (SPC) charts^24 25^ with the NHS Excel XmR (individuals) template,^26^ modified to display case sequence on the X axis rather than dates (see figure 2). We also monitored safety incidents (through the Datix reporting system) to check for any inadvertent safety issues.

Our chosen outcome metric (OM) was the delay experienced by the patient from their given appointment time to the time their treatment started. Baseline data (the 21 successive cases from September 2019 to September 2020) had an average delay of 52.4 min with considerable process variation (see figure 2, top). The upper process limit (UPL), which shows the expected maximum extent of random variation (mean+3 SDs), was around 3 hours. Our (internally agreed) target was to keep delays below 30 min. In 10 of these 21 cases, the treatment plan was not ready 30 min before the scheduled New Start time.

The physicists then conducted a root causes analysis^6^ to investigate causes of treatment delay at New Start caused by planning issues. shows an Ishikawa (cause-and-effect or fishbone) diagram^6 27^ of potential causes of these delays. This highlighted manual data entry and a lack of trained staff. To investigate these issues further, we analysed Datix incidents recorded over the same time period. shows a Pareto chart^5 6^ of these 13 incidents. The main causes were plan not ready and manual data entry (together causing over 90% of the incidents). These initial analyses motivated the QI project described in this paper.

To monitor the impact of changes on important parts of the planning pathway, we established a set of three process metrics (PMs), as shown on figure 1, with baseline values as shown on the SPC charts in figure 2:

PM1: the time taken to complete the initial planning by the radiographer, including any prior wait. The baseline was 46.0 hours, UPL nearly 160 hours.

PM2: the time taken to complete the Mould Room Checks, including any prior wait. The baseline was 64.9 hours, UPL over 250 hours.

PM3: the time taken to complete the Final Checks by the radiographer, as shown on the relevant parts of figure 1A,B, including any prior wait. The baseline time was 23.6 hours with UPL around 135 hours. One patient was an outlier, initially delayed due to waiting for a skin graft to heal and then further delayed due to our electron-capable treatment machines being moved from our Wirral site to our Liverpool site.

We set internal targets of 12 hours for the initial plan to be completed and 24 hours for each of Mould Room Checks and Final Checks to be completed.

## Design

Reviewing the data, the QI team considered the third question of the MfI:

*What changes can we make that will result in the improvements that we seek?*

### Change idea A: introduce a (proxy) day 0 appointment

This idea came about as a result of treatment plans not being ready on time (identified on the Pareto chart of online supplemental figure S1) and the risk of incorrect data entry identified in the Ishikawa (fishbone) diagram (online supplemental figure S2).

10.1136/bmjoq-2022-002221.supp1 Supplementary data

As explained in the Background section, because non-scanned electron patients do not receive a CT planning scan, a treatment simulation was required to obtain the optimal gantry, collimator and couch positions for radiation treatment. Prior to this project, this was done through a treatment simulation session at the patient’s New Start (ie, when the patient was present for their first radiotherapy treatment appointment). At this point, the approved plan would be unapproved so that it could be updated with the final treatment parameters. This step increased the likelihood of error, as it is possible to make inadvertent changes to the plan while it is in the unapproved state, adding to the pressure felt by staff. The plan would then be reapproved and radiotherapy treatment delivery could start.

If the patient has a thermoplastic cast, as nearly all electron patients do, the treatment area is drawn on the cast. The cast can be attached to the couch without the patient present. This means that the treatment simulation session (to determine the optimal gantry, collimator and couch positions) can be completed without the patient present. Then, at New Start, the patient can proceed directly to treatment and pressure on staff is reduced as they do not have to simulate treatment or unapprove the plan at this initial patient encounter.

Thus, the idea was to add a day 0 proxy appointment to implement this change. The very few electron patients (around 5%) who do not have a cast are required to be present for this appointment, but no dose is delivered.

We predicted this day 0 proxy appointment would reduce delays by ensuring that the plan is ready for treatment by the day prior to the patient arriving for their New Start treatment, so the patient can proceed directly to treatment at their appointment time.

### Change idea B: make the treatment planning on this care path (non-scanned electron patients) more similar to the dominant (photon) EBRT care paths

Because electron patients do not have a prior planning CT scan, their pathway required a manual calculation and manual check process to produce a ‘pseudo-plan’ in the ARIA radiotherapy record-and-verify system. It also required manual data entry. It was less familiar to staff and (so) more prone to error. This was unlike any other EBRT plan type—for which the plan is produced in the treatment planning system Eclipse^28^ and directly picked up by ARIA.

Change idea B was therefore to make this pathway more similar to the others by producing a pseudo-plan within Eclipse. This plan could then be checked prior to being handed over to radiographers. With this change, only the presence of day 0 makes this pathway different from the majority of our EBRT care planning paths.

### Change idea C: remove the TEP Sig step

The TEP Sig was a step after the first fraction of treatment (see figure 1)—a sign off being required for any pathway involving manual data entry and so at increased risk of introducing human error. However, it is a duplication and, following the removal of the manual data entry in change idea B, risk assessment showed that the check was now redundant.

## Strategy

Applying the MfI, we conducted PDSA cycles to test and refine the change ideas.

### PDSA on change idea A: introduce a (proxy) day 0 appointment

The first change, tested in PDSA cycle A1, was to introduce the day 0 appointment on the day before a New Start appointment. This change was introduced on 26 October 2020 and six patients were treated with day 0 as a floating appointment (ie, not attached to the care path). This change worked and as a result, all six patient plans were ready in time for their New Start and all six patients were treated within 30 min of their appointment time (see figure 2, top). However, some issues arose, primarily that the day 0 planning simulation took place after the Final Checks, so changes were made to the plan after these checks—which is not considered good practice. In addition, the necessary staff were not always aware of the day 0 appointment since the task was not connected to the care path.

A quick refinement was implemented to move day 0 to before Final Checks and attach it to the care path. This was tested with cycle A2. A number of incident reports were raised because the patient planning details had not been provided (by the Mould Room) and patients had been booked onto linacs without electron capability for their treatment course.

A refinement for cycle A3 was therefore to add a Mould Room Prep task early in the care path to ensure planning data would be available, and for information technology to make changes to the patient booking system to avoid incorrect bookings. At this point, good progress had been made, but it was noted that some tasks in the care path, notably Mould Room Checks and Final Checks, were taking a long time to complete (the latter was actually taking longer than previously).

Cycle A4 therefore started with staff engagement sessions, where it was suggested that day 0 was completed 2 days prior to New Start. This refinement was tested in this cycle.

This project started during COVID-19, when overall workload at CCC was lower than usual. As this general workload increased again, it became more difficult to find time to perform the day 0 appointment on our specific electron-capable linacs. Therefore, as a final refinement to change idea A, we obtained formal agreement to allow the day 0 tasks to be undertaken on any linac (cycle A5).

### PDSA on change idea B: make this care path more similar to most others

The idea was to remove manual calculations and data entry, instead producing a pseudo-plan within the Eclipse software. These changes (B1) were tested in conjunction with cycle A3. The impact on the process metrics was small; however, these changes had a larger impact on the safety of the planning process and incidents relating to manual data input for these patients were eradicated. An unintended consequence of the change was that the care planning paths were so similar that staff were unable to distinguish between non-scanned and scanned electron patients, which caused some confusion.

Cycle B2 refined the idea, therefore, by adding clarification information (clearly labelling scanned vs non-scanned electron patient) to the immobilisation documentation.

### PDSA on change idea C: remove now redundant TEP Sig step

To obtain permission for this, we prepared an updated risk assessment and had this agreed by the Quality and Safety Committee.

## Results

QI interventions began in October 2020. From then until the end of our study period, 75 non-scanned electron patients were treated. We have reduced the mean delay at New Start (figure 2, OM) from 52.4±45.5 min to 27.2±26.8 min. This is within our internal target of 30 min, but there remains considerable variation with a relatively large UPL, so our performance is not as reliable (consistent) as we would like.

Prior to the QI project, 33% of patients started their first fraction within 30 min of their appointment time; for the first 12 months of the project, this increased to 63.2%; and in the final cycle (A5), this further improved to 69.2%. However, there are confounding factors on these timings, such as other delays and machine breakdown. Therefore, we also analysed the impact of planning by considering the number of patient plans actually ready by 30 min prior to the New Start appointment time. Prior to the QI project, 48% of plans complied with this; in the final cycle, we improved this to 92.3%. We consider this a major achievement and benefit to patients.

Additionally, prior to this project, there were 10 potential safety (Datix) incidents recorded (from a sequence of 21 patients) due to plans not being ready by the New Start time. This has been reduced to zero as a result of the changes made during this project.

We have reduced the time to complete initial planning (PM1) from 46.0 hours to our internal target (12 hours), on average. We reduced delays at other stages through staff engagement sessions, for example, prior to the project, Mould Room Checks (PM2) took an average of 64.9±57.4 hours. Through our PDSA cycles, we have reduced this to 25.0±23.0 hours, close to our target of 24 hours. This gives us more safety margin later in the process.

It is disappointing to note that Final Checks (PM3) now takes longer to complete than it did prior to the improvement project, initially 23.6±47.8 hours vs 47.1±42.5 hours in cycle A5, and we are far from our 24-hour target. This may be an unintended consequence of the extra time noted above: going beyond safety margin to overly relaxed. We intend to share these data with staff, as was effective for Mould Room Checks, in an effort to reinforce awareness and improve Final Checks completion times.

Moving away from manual data entry in ARIA (cycle B1) did not feed through directly to reduced delays at New Start (our OM), contrary to our predictions. We thought that by making plans more similar to other EBRT sites, and so increasing the pool of trained staff available to work on the pathway, we would reduce delays. While the number of trained staff has increased, the overall treatment delays (OM) have remained fairly constant since the introduction of the day 0 appointment in cycle A1. Looking at figure 2 suggests that, as noted above, shortened initial planning duration (early in the planning process) was countered by lengthened duration for Final Checks (at the end). However, these changes to the pathway have improved the safety of the planning process.

### Sustainability

The change ideas have been adopted and sustained. As described above, the baseline was the full year of patients treated prior to our changes (ie, up to October 2020, ‘baseline’) and our final project evaluation was the last set of patients treated (ie, up to June 2022, ‘end of project’). To assess sustainability, we went back to look at the full year since (ie, to June 2023) on Datix and the most recent group of patients (n=10) treated May–June 2023 on timings (‘2023’) .

- The average delays to New Start were: baseline (52.4 min); end of project (27.2 min); 2023 (26.0 min).
- The proportion of patients treated within our target of no more than 30 min after their appointment times was: baseline (33%); end of project (69%); 2023 (50%).
- The proportion of patient plans ready 30 min before the appointment times was: baseline (48%); end of project (92%); 2023 (80%).
- Datix events due to plan not ready: baseline (10); end of project (0); 2023 (0) (in the *full year* of data (June 2022–June 2023)).

So the data indicate that we have sustained most of our gains. In the next section, we briefly discuss factors beyond our control which can delay starts; while the first two of the above metrics are most obvious to the patient, the second two are probably the best measure of success for our project.

## Lessons and limitations

Some changes were very successful—in particular the day 0 proxy appointment—but took several cycles of PDSA learning and refinement to embed effectively. Some of these refinements might have been built into the change idea design initially, but it is hard to foresee the impacts of such fundamental changes to a pathway like this.

As clear in table 1 and figure 2, in some PDSA cycles, we tested several different change ideas or refinements at the same time. This is not good QI practice as it makes the effects of different changes hard to disentangle.^29^ It was a compromise here due to the need to make progress in attempting improve performance and maintain QI momentum, even though data (cases) build up relatively slowly in this system.

**Table 1:** PDSA cycles for improvements to the electron (non-scanned) patient treatment care path

Cycle	Plan/predictions	Do	Study (data are mean±SD)	Act
Change idea A: introduce day 0				
A1	Introduce day 0 appointment after Final Checks **Predictions:** Reduced pressure on staff Improved safety Improved ability to deliver New Start on time	26 Oct 2020 Amend care path on ARIA Make staff aware	OM: 11.3±11.6 min, 100% of plans ready on time and 100% of patients treated within 30 min of appointment time PM1 28.0±31.5 hours PM2 83.2±58.6 hours PM3 8.7±1.0 hours Staff not aware of day 0 appointment	Change retained **Further improvements required**: Final Checks should be after day 0 Attach day 0 to care path Raise staff awareness
A2	Move Final Checks to after day 0 Add staff as a resource to day 0 task **Predictions:** Improved safety & staff availability	01 Jan 2021 Amend care path on ARIA Check and add to care path Make staff aware	Incidents recorded: 2 booked onto incorrect machine 1 no day 0 appointment booked (Mould Room task) 2 no immobilisation document (Mould Room task) 1 no photos present (Mould Room task)	Change retained **Further improvements required:** Ensure Mould Room tasks completed prior to planning Avoid booking patients onto incorrect machine
A3	Add in additional Mould Room task Remove possibility for booking onto incorrect machine **Predictions:** Reduced delays at planning & incorrect bookings	01 Mar 2021 Amend care path on ARIA Contact IT to change booking system to only appropriate linac	OM: 19.6±30.3 min, 67% of plans ready on time and 75% of patients treated within 30 min of appointment time PM1 11.0±18.9 hours PM2 59.4±46.8 hours PM3 31.2±66.6 hours	Change retained Pinch points revealed in completion time data **Further improvements required**: Move day 0 appointment earlier
A4	Move day 0 to 2 days prior to treatment **Predictions:** Improved number of plans completed in time for New Start	28 June 2021 Amend care path on ARIA Booking Desk and Mould Room informed	OM: 26.1±27.6 min, 72% of plans ready on time and 61.4% of patients treated within 30 min of appointment time PM1 17.0±21.7 hours PM2 26.9±24.5 hours PM3 59.5±40.5 hours	Change retained **Further improvements required**: Electron-capable machines were seeing high usage, other linacs less heavily used. To maintain benefits of day 0: Allow it to be completed on any linac
A5	Allow day 0 task on any linac **Predictions:** Benefits of day 0 would be maintained, while not impacting on availability of patient appointments, despite rising workload	12 April 2022 Obtain permission (instead of under concession): work instructions updated Evaluate sustainability as COVID-19 eases	OM: 27.2±26.8 min; 92.3% of plans ready on time and 69.2% of patients treated within 30 min of appointment time PM1 12.0±13.5 hours PM2 25.0±23.0 hours PM3 47.1±42.5 hours	Performance maintained Change retained
Change idea B: make pathway more like other EBRT treatments				
B1	Remove manual calculations and data input **Predictions:** Reduced staff’s lack of familiarity and stress, improved safety	01 Jan 2021 Implement pseudo-plan in Eclipse Plan checking questionnaires Write and distribute staff training and work instructions	No Datix incidents Care path for scanned and non-scanned electrons now similar→confusion as to which is which!	Change retained **Further improvements required**: Need to be able to identify plan type
B2	Add additional identification of scanned/non-scanned patient **Predictions:** Improved plan-‘type’ identification	31 Aug 2021 Additional clarification on immobilisation document Additional patient alerts Additional staff training	Changes are made for safety not efficiency—metrics not recalculated	Change retained
Change idea C: remove TEP Sig task				
C	Remove TEP Sig step (now redundant) **Predictions:** Can remove TEP without issues, and so free some staff time	31 Aug 2021 Prepare risk assessment and share with Quality and Safety Committee Ensure all tasks covered elsewhere Update tick lists Remove TEP Sig task	With B2, and a downstream of New Start—metrics not recalculated	No issues detected Change retained

EBRT, external beam radiotherapy; IT, information technology; OM, outcome metric; PDSA, Plan–Do–Study–Act; PM, process metric; TEP Sig, treatment expert practitioner sign-off.

Our aim was to reduce delays at New Start, as a proxy for improving patient experience. While by the end of the project, our treatment plan was now ready for over 92% of patients (vs 48% previously), we were at 69% of New Starts within 30 min of appointment time (vs 33% previously)—some way short of our aspiration of 90%.

### Confounding factors

Prior to this project, we predicted that removing the manual calculation and data entry into ARIA would directly feed through to reduced delays at New Start, but this was not the case. We found Final Checks were taking longer than they did prior to the improvement project and concluded we had not anticipated the reaction of staff to the consequential increased breathing space towards the end of the planning pathway, and we found that staff awareness and engagement sessions had a material effect on the time taken to complete some tasks and it may be useful to repeat and reinforce this for the Final Checks task. Anticipation of this risk, and the value of focusing staff awareness, is a learning point.

As noted, the aim of our project was to contribute to patient experience through reduced delayed New Starts, and this was our OM. There are other causes of delays, including machine breakdown (leading to delays while the machine is repaired or cancellation of the session), the patient themselves arriving late or staffing issues. These were exogenous (or confounding) factors out of scope of our project and we did not measure them. In retrospect, we could have thought more clearly about these; a driver diagram^5 30^ may have helped make this explicit. We might then have decided to set a different direct aim for the project, such as focusing on plan-ready time, and/or also recorded and analysed exogenous causes of delay as balancing metrics. These other factors are potential targets for future QI projects.

## Conclusion

This project aimed to improve patient experience through the proxy measure of reducing the delays they experience at their first radiotherapy treatment (New Start), inevitably anyway a stressful occasion. Consequent secondary outcomes were to reduce staff stress at New Start and to improve the safety of the hitherto rather manual and less familiar electron treatment planning pathway.

Our initial data collection showed that the majority of patients were experiencing material delays at New Start and that, in the majority of cases, plans were not ready in time for treatment. There had been previous attempts to improve workflow, but these focused on individual tasks rather than the entire pathway.

We judge the project a success: our change ideas have proved worthwhile. Delays at New Start have been reduced very considerably, and in particular the contribution of treatment planning to this. In addition, the non-scanned electron radiotherapy planning pathway is now better aligned with our other EBRT pathways at CCC, with unfamiliar and risk-prone manual tasks removed. This has enabled an increased pool of trained staff available to work on the pathway and has increased patient safety.

## Data Availability

All data relevant to the study are included in the article or uploaded as supplemental information.

## References

[1] The Clatterbridge Cancer Centre. The Clatterbridge cancer centre NHS foundation trust. Available: www.clatterbridgecc.nhs.uk/about-centre/mission-aims-and-values [Accessed 26 Jun 2023].

[2] Hoskin P, Coyle C. Radiotherapy in practice - Brachytherapy. Oxford: Oxford University Press, 2011.

[3] Hoskin P, ed. Radiotherapy in practice - External beam therapy. 3rd ed. Oxford: Oxford University Press, 2019. 10.1093/med/9780198786757.001.0001

[4] Boaden R, Harvey G, Moxham C, et al. Quality improvement: theory and practice in healthcare. Coventry: NHS Institute for Innovation and Improvement, 2008. Available: www.england.nhs.uk/improvement-hub/wp-content/uploads/sites/44/2017/11/Quality-Improvement-Theory-and-Practice-in-Healthcare.pdf

[5] Langley GJ, Moen RD, Nolan KM, et al. The improvement guide: a practical approach to enhancing organizational performance. 2nd ed. San Francisco: Wiley, 2009.

[6] Elkhuizen S, Proudlove N. Chapter 6: improvement approaches. In: Vissers J, Elkhuizen S, Proudlove N, eds. Operations management for healthcare. 2nd edn. Abingdon, UK: Routledge, 2023: 123–41. 10.4324/9781003020011

[7] Li Y, Proudlove N. A quality improvement project to reduce the turnaround times of infectious disease markers reporting in an NHS stem cell department. BMJ Open Qual 2022;11:e001814. 10.1136/bmjoq-2022-001814 PMC9196163 35697357

[8] White E, Proudlove N, Kallon D. Improving turnaround times for HLA-B*27 and HLA-B*57:01 gene testing: a Barts health NHS trust quality improvement project. BMJ Open Qual 2021;10:e001538. 10.1136/bmjoq-2021-001538 PMC8438818 34518303

[9] May F, Pepperall J, Davies E, et al. Summarised, verified and accessible: improving clinical information management for potential haematopoietic stem cell transplantation patients. BMJ Open Qual 2021;10:e001605. 10.1136/bmjoq-2021-001605 PMC8543752 34686487

[10] McCullagh J, Proudlove N, Tucker H, et al. Making every drop count: reducing wastage of a novel blood component for transfusion of trauma patients. BMJ Open Qual 2021;10:e001396. 10.1136/bmjoq-2021-001396 PMC8268902 34244177

[11] Pridgeon M, Proudlove N. Getting going on time: reducing neurophysiology setup times in order to contribute to improving surgery start and finish times. BMJ Open Qual 2022;11:e001808. 10.1136/bmjoq-2021-001808 PMC9310250 35863774

[12] Corner C, Tharmalingam H, Hoskin P. Skin cancer. Radiotherapy in practice - external beam therapy. 3rd edn. Oxford: Oxford University Press, 2019: 438–53.

[13] EFOMP. Policy statement NR 9: radiation protection of the patient in Europe: the training of the medical physics expert in radiation physics or radiation technology. 1999. Available: www.efomp.org/uploads/policy_statement_nr_9.pdf [Accessed 26 Jun 2023].

[14] James H, Hodges G, Tharmalingam H. Ionising radiation (medical exposure) regulations: implications for clinical practice in radiotherapy. The Radiotherapy Board (made up of the Society and College of Radiographers,Institute of Physics and Engineering in Medicine and The Royal College of Radiologists). Produced in association with Public Health England, 2020. Available: www.rcr.ac.uk/sites/default/files/guidance-on-irmer-implications-for-clinical-practice-in-radiotherapy.pdf [accessed 26 Apr 2021].

[15] Varian. ARIA oncology information system. Varian: A Siemens healthineers company 2023. Available: www.varian.com/en-gb/products/software/information-systems/aria-oncology-information-system [Accessed 26 Jun 2023].

[16] RLDatix. Event reporting. 2022. Available: https://rldatix.com/en-nam/solutions/how-we-help/risk/event-reporting/ [Accessed 26 Jun 2023].

[17] MED. What happens when I submit a Datix? The medical education directorate, NHS Lothian. Available: www.med.scot.nhs.uk/trainee-doctors/learning-from-datixes/what-happens-when-i-submit-a-datix#:~:text=What%20is%20DATIX%3F,identify%20learning%20and%20implement%20improvement [Accessed 26 Jun 2023].

[18] Munshi A, Krishnakutty S, Sarkar B, et al. Daily waiting and treatment times at an advanced radiation oncology setup: a 4-year audit of consecutive patients from single institution. J Cancer Res Ther 2021;17:523–9. 10.4103/jcrt.JCRT_685_19 34121702

[19] Damato AL, Lee LJ, Bhagwat MS, et al. Redesign of process map to increase efficiency: reducing procedure time in cervical cancer Brachytherapy. Brachytherapy 2015;14:471–80. 10.1016/j.brachy.2014.11.016 25572438 PMC4468005

[20] Vitzthum L, Yuan J, Jones D, et al. Reducing prolonged chemoradiation treatment times for cervical cancer. BMJ Open Qual 2019;8:e000516. 10.1136/bmjoq-2018-000516 PMC6768377 31637317

[21] Price G, Devaney S, French DP, et al. Can real-world data and rapid learning drive improvements in lung cancer survival? The RAPID-RT study. Clinical Oncology 2022;34:407–10. 10.1016/j.clon.2021.12.017 35000827

[22] Trebble TM, Hansi N, Hydes T, et al. Process mapping the patient journey: an introduction. BMJ 2010;341:c4078. 10.1136/bmj.c4078 20709715

[23] Rother M, Shook J. Learning to see: value-stream mapping to create value and eliminate muda. 1.3 ed. Brookline, MA: The Lean Enterprise Institute, 2003.

[24] Mohammed MA, Worthington P, Woodall WH. Plotting basic control charts: tutorial notes for healthcare practitioners. Qual Saf Health Care 2008;17:137–45. 10.1136/qshc.2004.012047 18385409

[25] Provost LP, Murray SK. The health care data guide: learning from data for improvement. 2nd ed. San Francisco, CA: John Wiley and Sons, 2022.

[26] NHS England. Statistical process control tool. 2021. Available: www.england.nhs.uk/statistical-process-control-tool [Accessed 20 Mar 2021].

[27] Best M, Neuhauser D. Kaoru Ishikawa: from Fishbones to world peace. Qual Saf Health Care 2008;17:150–2. 10.1136/qshc.2007.025692 18385411

[28] Varian. Eclipse. Varian: a Siemens Healthineers company 2023. Available: https://www.varian.com/en-gb/products/radiotherapy/treatment-planning/eclipse [Accessed 28 Jun 2023].

[29] Leis JA, Shojania KG. A primer on PDSA: executing plan–do–study–act cycles in practice, not just in name. BMJ Qual Saf 2017;26:572–7. 10.1136/bmjqs-2016-006245 27986900

[30] Bennett B, Provost L. What’s your theory? Driver diagram serves as tool for building and testing theories for improvement. Quality Progress 2015;48:36–43.

