# Supplementary material for: Reducing first appointment delays for electron radiotherapy patients by improving the treatment planning pathway: a quality improvement project
Source: BMJ Open Qual. 2023 Nov 21;12(4):e002221. doi: 10.1136/bmjoq-2022-002221 (PMC10668264; doi:10.1136/bmjoq-2022-002221)
Supplement: Supplementary data [file bmjoq-2022-002221supp001.pdf]

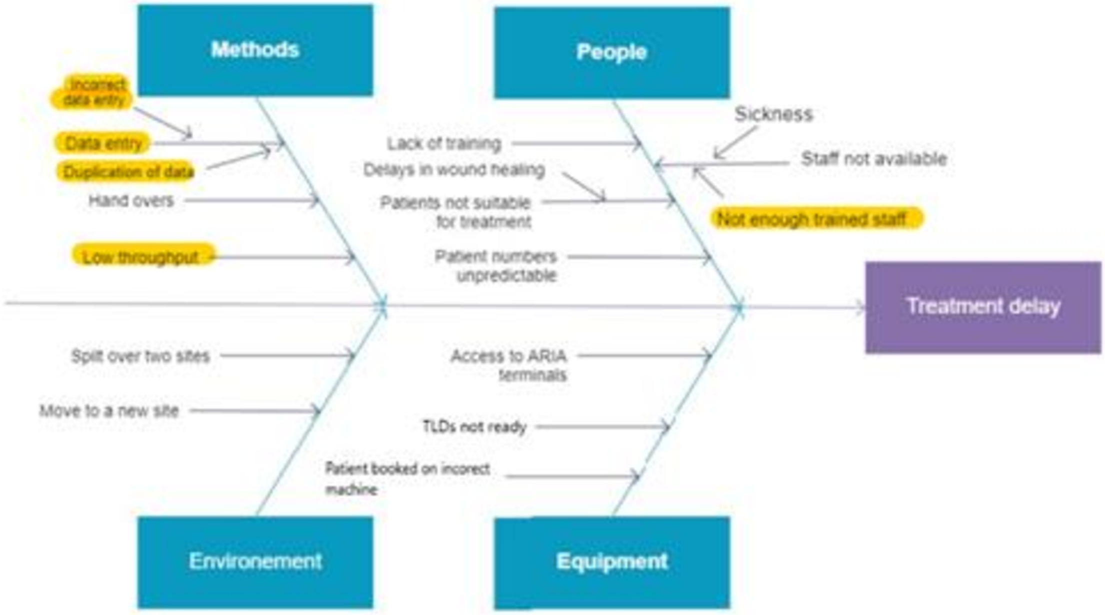

Figure S1: Cause and effect (fishbone) diagram showing delays in pathway

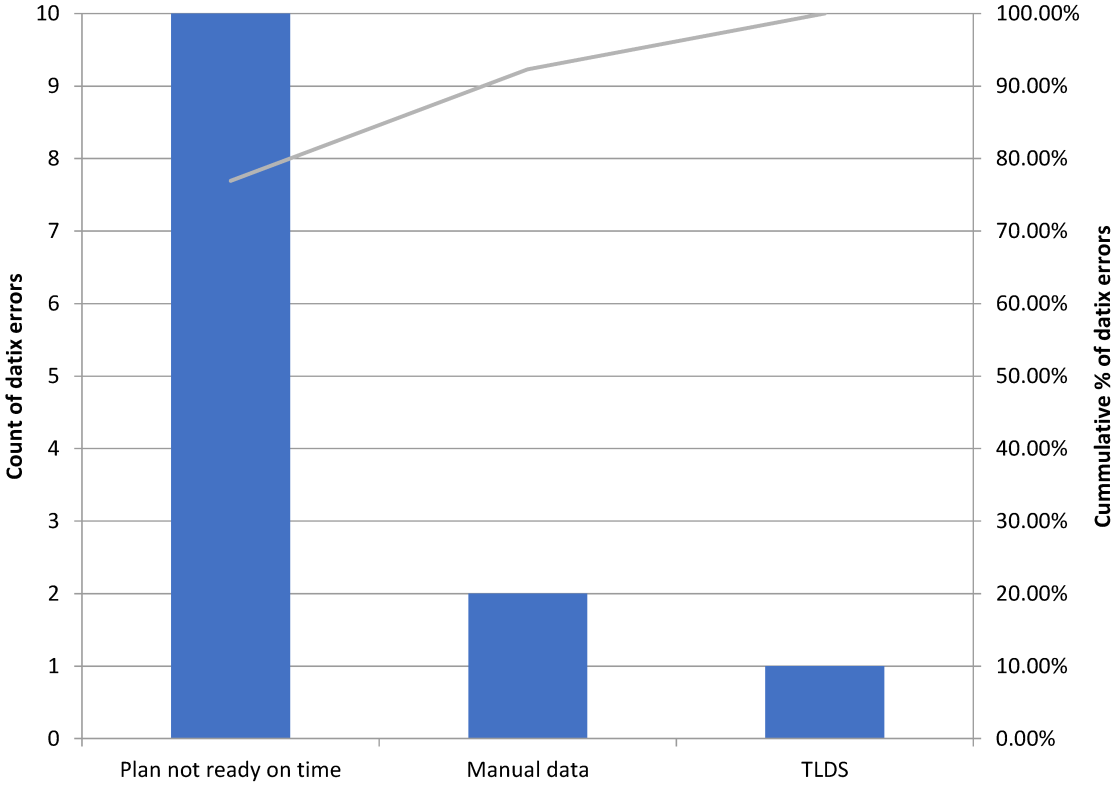

Figure S2: Pareto chart of Datix incidents split by category – baseline n=21 patients
